# Burden of Respiratory Syncytial Virus Infection in Children and Older Patients Hospitalized with Asthma: A Seven-Year Longitudinal Population-Based Study in Spain

**DOI:** 10.3390/v16111749

**Published:** 2024-11-07

**Authors:** Rosa María Gomez-Garcia, Rodrigo Jiménez-Garcia, Ana López-de-Andrés, Valentín Hernández-Barrera, David Carabantes-Alarcon, José J. Zamorano-León, Natividad Cuadrado-Corrales, Ana Jiménez-Sierra, Javier De-Miguel-Diez

**Affiliations:** 1 Respiratory Care Department, Hospital General Universitario Gregorio Marañón, Instituto de Investigación Sanitaria Gregorio Marañón (IiSGM), Universidad Complutense de Madrid, 28007 Madrid, Spain; rosamg07@ucm.es (R.M.G.-G.); javier.miguel@salud.madrid.org (J.D.-M.-D.); 2 Department of Public Health & Maternal and Child Health, Faculty of Medicine, Universidad Complutense de Madrid, 28040 Madrid, Spain; rodrijim@ucm.es (R.J.-G.); dcaraban@ucm.es (D.C.-A.); josejzam@ucm.es (J.J.Z.-L.); mariancu@ucm.es (N.C.-C.); 3 Department of Public Health & Maternal and Child Health, Faculty of Pharmacy, Universidad Complutense de Madrid, 28040 Madrid, Spain; 4 Preventive Medicine and Public Health Teaching and Research Unit, Health Sciences Faculty, Rey Juan Carlos University, Alcorcón, 28922 Madrid, Spain; valentin.hernandez@urjc.es; 5 Faculty of Medicine, Universidad San Pablo Ceu, 28668 Madrid, Spain; a.jimenez100@usp.ceu.es

**Keywords:** respiratory syncytial virus, asthma, children, elderly patients, hospital admissions

## Abstract

(1) Background: To describe hospitalizations due to respiratory syncytial virus (RSV) infection among children and elderly patients with asthma. (2) Methods: We used a nationwide discharge database to select patients with asthma aged 0 to 15 years and ≥65 years admitted to Spanish hospitals from 2016 to 2022. (3) Results: We identified 49,086 children and 471,947 elderly patients hospitalized with asthma (3.52% and 0.51%, respectively, with RSV). The proportion of RSV increased over time in children with asthma (from 1.44% to 7.4%, *p* < 0.001) and in elderly individuals (from 0.17% to 1.01%, *p* < 0.001). Among children with RSV infection, the presence of influenza (OR 3.65; 95% CI 1.46–9.1) and pneumonia (OR 1.85; 95% CI 1.02–3.55) increased the risk of poor outcome. The presence of RSV was associated with severity in these patients, defined by use of mechanical ventilation and/or admission to the intensive care unit (OR 1.44; 95% CI 1.11–1.86). In elderly patients with RSV infection, older age, congestive heart failure, COVID-19, and pneumonia increased the risk of in-hospital mortality (IHM). However, RSV infection was not associated with IHM (OR 0.88; 95% CI 0.68–1.15) in these patients. (4) Conclusion: Our results highlight the impact of RSV infection in children and elderly patients hospitalized with asthma. Strategies to improve surveillance, prophylaxis, and management of RSV infection should be evaluated.

## 1. Introduction

Respiratory syncytial virus (RSV) is a ubiquitous respiratory virus that belongs to the *Pneumoviridae* family, genus *Orthopneumoviridae*. There are two subgroups (A and B), differing from each other in their molecular structure [1]. RSV transmission occurs via large droplet inoculation in the eyes, nose, or mouth, requiring close contact with an RSV-infected subject or auto-inoculation to the face (nose, mouth, or eyes) via contaminated fomites or skin [2]. Like other respiratory viruses, RSV infection results in annual recurring events (seasonal epidemics) [1]. In Spain, according to the 2021–2022 season, RSV infections produced approximately one million primary care consultations. On the other hand, there were 23,000 hospitalizations due to RSV in such season. These data indicate that RSV infection is very frequent and translates into a high healthcare burden in Spain [3].

It presents a range of acute respiratory tract infections, with severity varying from mild upper respiratory symptoms to severe lower respiratory tract infections and complications [4,5]. Populations most vulnerable to severe RSV infection include children, particularly premature infants, elderly individuals, and those with underlying pulmonary conditions [6].

RSV is the primary pathogen linked to hospital admissions for lower respiratory tract infections in children [7] and represents a significant contributor to morbidity and mortality in infants [8]. Evidence from multiple studies suggests an association between severe early RSV infection and an elevated risk of asthma development in later childhood [9,10]. Although the precise nature of this relationship remains unclear [11], RSV-induced persistent inflammation and airway hyperreactivity are likely consequences of alterations in both local and systemic immune responses, alongside structural airway changes that may occur concurrently or at distinct stages [12]. Additionally, RSV infection plays a crucial role in the exacerbation and progression of asthma [13], affecting individuals across both childhood and adulthood [14].

In adults, symptoms of RSV infection are usually absent or mild. However, in immunocompromised patients, adults with chronic cardiopulmonary diseases, and the elderly, RSV can cause severe lower respiratory complications, resulting in respiratory failure, prolonged hospitalization, and high mortality [15,16]. In fact, several systematic reviews have highlighted the considerable clinical burden of RSV infection in such patients [17,18,19].

RSV is a common trigger of acute asthma exacerbation. A series of typical pathological characteristics (including more marked airway inflammation, increased airway hypersensitivity, and severe airway obstruction) are induced in patients with asthma who experience acute exacerbation after RSV infection, thus contributing to the rising incidence and hospitalization rates in this population [20]. Therefore, more research is needed to clarify the relationship between RSV and asthma exacerbation.

Several novel preventive approaches have recently become available, including monoclonal antibodies for preventing RSV infection in high-risk pediatric populations, and vaccines recently approved for use in pregnant women (to provide passive immunity in infants) and elderly patients [5,21,22]. Robust epidemiological data are crucial for the effective deployment of these new preventive strategies [23,24]. Enhanced understanding of the real-world RSV burden among these patient groups is valuable for informing public health policymakers and for optimizing RSV infection management and prevention efforts [23], particularly in individuals with comorbidities such as asthma.

The objectives of our study were to describe hospitalizations due to RSV infection in the Spanish public healthcare system between the years 2016 and 2022. We specifically analyzed individuals with asthma considered vulnerable, namely, children and elderly persons. We recorded demographic characteristics, comorbidities, symptoms, use of therapeutic procedures, length of hospital stay (LOHS), costs, in-hospital mortality (IHM), and severity. Finally, we compared hospitalization outcomes between children and elderly patients with asthma and RSV infection and age- and sex-matched individuals with asthma but without RSV infection.

## 2. Materials and Methods

We designed a descriptive observational study. The data for this study were obtained from the Spanish Hospital Discharge Database (SHDD), which is managed by the Ministry of Health. By law, all hospitals must submit information for all hospitalized patients, including basic demographic characteristics, the principal diagnosis leading to admission, up to 19 secondary diagnoses (present at admission or diagnosed during the hospital stay), up to 20 diagnostic or therapeutic procedures performed during the hospital stay, admission to the intensive care unit (ICU), the outcome of hospitalization (discharge, death), and costs. Details of the SHDD are available online [25]. The coding of diagnoses and procedures in the SHDD is based on the International Classification of Diseases, 10th Revision (ICD-10). We used SHDD data from 2016 to 2022.

### 2.1. Study Population

The study population consisted of individuals aged 0 to 15 years (children) and 65 years and older (elderly) who were hospitalized during the study period in public hospitals in Spain with a diagnosis of asthma, either as the primary or secondary diagnosis (ICD-10 code J45). We excluded persons for whom data on age, sex, LOHS, or hospitalization outcome were missing. To identify children and elderly patients with RSV, we searched for ICD-10 codes J12.1, J20.5, J21.0, and B97.4 in any diagnostic field. Two populations were defined: individuals with asthma and RSV infection and individuals with asthma but without RSV. To obtain comparable populations and analyze the effect of RSV infection on hospitalization outcomes, each individual with RSV was matched with an individual without RSV based on the asthma diagnostic position (1 to 20), year of admission, age, and sex. If more than one person without RSV infection met these criteria, participants were selected randomly.

### 2.2. Study Variables

The primary outcome variables were the use of mechanical ventilation (invasive or non-invasive), ICU admission, LOHS, and costs. For children, a variable defined as “severity” was created, including those who required any form of mechanical ventilation and/or ICU admission. IHM was not analyzed for children because no deaths among children diagnosed with RSV infection during the 2016–2022 period were recorded. For elderly patients, IHM was analyzed. 

The covariates included sex, age, comorbidities present on admission or diagnosed during hospitalization, and symptoms and signs of RSV infection. In elderly patients, comorbidity was quantified using the Charlson Comorbidity Index (CCI) for ICD-10 administrative data following recommendations from other authors [26,27]. The ICD-10 codes for the diagnoses and procedures analyzed are provided in Table S1.

### 2.3. Statistical Analysis

For the description of the study population, absolute and relative frequencies are presented as percentages for qualitative variables and as means (standard deviation) or medians (interquartile range) for quantitative variables. Trends over time from 2016 to 2022 for qualitative variables were analyzed using the Cochran–Armitage test or the Cochran–Mantel–Haenszel statistic, while a linear regression *t*-test or the Jonckheere–Terpstra test was used for quantitative variables. Proportions were compared using Fisher’s exact test. For quantitative variables, we applied the *t*-test or, if the distribution was not normal (according to the Kolmogorov–Smirnov test), we applied the Mann–Whitney test. 

Multivariable logistic regression models were constructed to control for confounding factors and identify which study variables were independently associated with severe outcomes in children or IHM in elderly patients. The variables included in the models were those for which a statistically significant association was recorded in the bivariate analysis, namely, sex, age, and year. The methodology used for the construction of multivariable models and for assessing collinearity and interaction between variables was as described by Hosmer et al. [28]. Odds ratios (ORs) with 95% confidence intervals (CIs) were obtained. Stata version 14 (Stata, College Station, TX, USA) was the statistical software used, and a *p*-value < 0.05 (2-tailed) was considered significant.

### 2.4. Ethical Considerations

The SHDD is owned by the Ministry of Health, which provides the databases to researchers upon evaluation of their proposals from a scientific and ethical perspective [29]. As it is an administrative database, Spanish legislation does not require informed consent from participants or an ethics committee report [25,29].

## 3. Results

A total of 49,086 children were hospitalized with asthma during the period from 2016 to 2022; of these, 1728 (3.52%) were diagnosed with RSV infection. Among the elderly, 471,947 individuals with asthma were hospitalized, with 2389 (0.51%) having an RSV code.

### 3.1. Temporal Trends in the Incidence and Characteristics of Children and Elderly Persons with Asthma and RSV

As shown in Table 1, the number of children with asthma and RSV infection increased steadily from 2016 to 2022 (from 121 to 515), except for 2020, where a significant decline was observed (119 cases). The frequency of RSV infection among children with asthma increased significantly, from 1.44% in 2016 to 7.4% in 2022 (*p* < 0.001). Boys accounted for 57.93% of the children, with no significant change in this proportion during the study period. The mean age increased from 2.09 years in 2016 to 3.43 years in 2022 (*p* < 0.001). The proportion of younger children (0–1 years) decreased over time, while those aged 2–5 years increased (*p* < 0.001). Children aged 0–1 years represented 41.05% of the population.

Invasive mechanical ventilation was very infrequent, recorded in only nine children between 2019 and 2022 (0.52%), while non-invasive ventilation was more common (4.51%) and remained stable over time. ICU admission was required in 7.35% of the children, and 9.55% were classified as severe cases, with no significant changes in these two variables during the study period. The median LOHS decreased significantly from 5 days in 2016 to 4 days in 2022 (*p* < 0.001). The cost per child hospitalized was €2916, with no significant temporal trend (*p* = 0.118).

Among the elderly, the number of hospitalizations for asthma and RSV infection increased eight-fold between 2016 and 2022 (93 vs. 779), with a significant increase in prevalence (0.17% to 1.01%; *p* < 0.001). In 2020, the number of hospitalizations dropped to only 123. Unlike children, elderly men accounted for only one in six cases, with no change between 2016 and 2022. The mean age increased from 78.68 years to 80.79 years over time (*p* = 0.006). Among the elderly, the use of invasive mechanical ventilation (1.72%; *p* = 0.169) and non-invasive ventilation (4.69%; *p* = 0.228) did not vary during the study period. The frequency of admission to the ICU decreased from 11.83% in 2016 to 2.7% in 2022 (*p* < 0.001). However, the number of deaths increased from two in 2019 (2.15%) to 39 (5.01%) in 2022, although this temporal trend was not statistically significant (*p* = 0.278). LOHS and costs did not change significantly between 2016 and 2022 (Table 1).

### 3.2. Comparison of Characteristics and Hospitalization Outcomes for Children and Elderly Persons with Asthma and RSV Infection Compared with Persons with Asthma Without RSV

After matching, it was found that children with asthma and RSV infection more frequently required non-invasive mechanical ventilation (4.52% vs. 2.43%; *p* < 0.001), were more often classified as severe (9.55% vs. 6.66%; *p* = 0.002), and had longer LOHS and higher costs than children with asthma who did not have RSV (Table 2).

In the elderly population, both invasive and non-invasive mechanical ventilation were more frequently coded in those with RSV infection than in those without (1.72% vs. 0.92%; *p* = 0.016 and 4.69% vs. 2.09%; *p* < 0.001, respectively). No differences were found in the percentage requiring admission to the ICU (4.48% vs. 3.93%; *p* = 0.349), IHM (5.53% vs. 6.74%; *p* = 0.080), or costs. LOHS was longer in elderly patients with RSV infection (median, 7 days vs. 6 days; *p* = 0.007).

### 3.3. Comparison of Underlying Conditions and Diagnoses Related in Children and Elderly Persons with Asthma and RSV Infection Compared with Persons with Asthma Without RSV Infection 

As shown in Table 3, the most frequently coded diagnoses related in children with asthma and RSV infection were acute bronchitis and bronchiolitis (11.23% each), which were significantly more prevalent than in children without RSV infection (*p* < 0.001 for both). However, pneumonia was less common (4.46% vs. 8.28%; *p* < 0.001), as were influenza and COVID-19 infection (Table 3). The prevalence of all underlying conditions was very low (<1%) in both populations, with no significant differences.

When elderly patients with asthma were matched based on the presence of RSV infection, it was observed that those without RSV had more comorbidities according to the CCI (1.56 vs. 1.35; *p* < 0.001) and, specifically, had more myocardial infarction, peripheral vascular disease, cerebrovascular disease, cancer, and COPD (Table 4). Among related diagnoses, acute bronchitis was the most frequently coded in elderly patients with RSV infection and was more common than in those without this infection (16.99% vs. 4.06%; *p* < 0.001). COVID-19 was more often coded in elderly patients without RSV infection, but the opposite occurred for influenza (Table 4). The presence of pneumonia did not differ between the groups with and without RSV infection (7.45% vs. 8.33%; *p* = 0.260).

### 3.4. Factors Associated with Severe Outcomes in Children and IHM in Elderly Patients: Multivariable Analysis 

Table 5 presents the ORs obtained in the multivariable logistic regression analysis performed to identify variables associated with severe outcomes in children with asthma based on the presence of RSV infection. Among children with RSV infection, only the presence of influenza (OR 3.65; 95% CI 1.46–9.1) and pneumonia (OR 1.85; 95% CI 1.02–3.55) increased the risk of poor outcome. Among children without RSV infection, variables associated with severe outcomes included acute bronchitis, bronchiolitis, and COVID-19.

When analyzing all children with asthma, it was observed that, after adjusting for other variables, the presence of RSV infection was significantly associated with severity (OR 1.44; 95% CI 1.11–1.86).

In elderly patients with asthma, the variables associated with IHM are shown in Table 6. Older age, congestive heart failure, COVID-19, and pneumonia increased the risk of IHM among those hospitalized with RSV infection. In addition to the variables mentioned, myocardial infarction and chronic kidney disease were also associated with IHM among participants without RSV infection. RSV infection was not significantly associated with IHM (OR 0.88; 95% CI 0.68–1.15) when the entire elderly population with asthma was analyzed.

## 4. Discussion

Our results highlight the impact of RSV infection in children and elderly patients hospitalized with asthma. We found that the number of children with asthma and RSV infection increased steadily in Spain from 2016 to 2022, with a notable decrease in 2020, probably due to public health interventions aimed at mitigating the COVID-19 pandemic, and an increase in subsequent years, as has been consistently reported in the scientific literature [30,31]. Our analysis also showed that the proportion of RSV infection among children with asthma grew significantly from 2016 to 2022. Possible explanations for this increase could include more widespread testing and underdiagnosis in earlier years, although we do not have data in our country to confirm this hypothesis. We also detected an age shift in the diagnosis of RSV infection, namely, that it was increasingly common in older children, as confirmed elsewhere [32,33,34]. Moreover, our research showed a clear predominance of male patients. In a recent study that estimated the rates of hospital admissions for RSV infection before the age of 2 years in England, Fonseca et al. reported that infants in the RSV-coded cohort were more likely to be male [35], consistent with previously published data in other countries. Anatomic and physiologic differences in the pediatric airway between males and females could explain these differences [36]. Evidence suggests that peripheral airways are disproportionately narrower in males during early life, potentially predisposing them to lower respiratory tract infections [37].

We found that children with asthma and RSV infection had a longer LOHS, required more non-invasive mechanical ventilation, and were more likely to be classified as severe than children with asthma without RSV. Cai et al. also recorded higher levels of RSV-related disease severity and a greater need for respiratory support [38]. Furthermore, Coutts et al. reported that hospitalization with RSV infection was associated with significantly increased rates and severity of asthma throughout childhood, thus highlighting the importance of preventive strategies [39].

Research on the economic impact of RSV infection among vulnerable infant populations is limited. In our study, costs were higher for children with asthma and RSV infection than for children with asthma without RSV infection. In this context, Armand et al. evaluated the incremental cost differences between patients with and without RSV infection, finding that RSV infection is associated with increased resource utilization and higher costs [40]. Beyond the direct costs of the RSV event, these findings may be attributed to complications, exacerbations of underlying conditions, and the long-term effects thought to stem from RSV infection [40].

Our investigation showed that the most frequently coded related diagnoses in children with asthma and RSV infection were acute bronchitis and bronchiolitis, both of which were significantly more prevalent than in children without RSV, while pneumonia and otitis were less common, as were influenza and COVID-19 infection. Vila et al. recently described the epidemiological, clinical, and virological features of pediatric hospitalizations for viral lower respiratory tract infections [41], reporting that influenza A was associated with pneumonia and required longer hospital stays, while RSV infection was associated with bronchiolitis and was the most frequent reason for admission to the pediatric ICU and for respiratory support. On the other hand, our study revealed that influenza and pneumonia increased the risk of poor outcome among children with RSV infection. Haeberer et al. have also shown, in children with RSV, that co-infection is a risk factor for hospitalization and severity [42].

Despite its substantial disease burden in adults, RSV is predominantly recognized as a pediatric pathogen. We observed very few RSV diagnoses among elderly patients. The disease burden in adults may be underestimated due to the absence of routine testing in this population, limited provider awareness, and the frequent oversight of RSV in infections leading to acute exacerbations of pre-existing respiratory conditions [43,44].

A fact that draws attention in our study is the imbalance of elderly men and women in patients with asthma and RSV. The marked difference between sexes has been widely studied in adult patients hospitalized with a diagnosis of asthma. Thus, it has been described that, in adulthood, women are three times more likely than men to be hospitalized for an asthma-related event. This difference has been linked to the regulatory activity of sex hormones in the pathophysiology of asthma, although the mechanism has not yet been clearly established [45,46].

In addition to older age, comorbidities have been described as important risk factors for hospital admissions with RSV infection in the adult population. Osei-Yeboah et al. reported that hospitalization rates for RSV infection were higher in adults with comorbidities than in the general population of the same age [47]. However, in our study, the mean CCI was lower in patients with RSV infection than in those without RSV infection. On the other hand, our results showed a significant increase in the number of elderly patients hospitalized with asthma and RSV infection from 2016 to 2022, with a decline in 2020, likely due to adherence by older adults to public health infection preventive measures during the COVID-19 pandemic [48,49]. In any case, the increases observed over time seem more likely to be due to broader access to polymerase chain reaction assays with a viral panel than to real epidemiological modifications, as suggested by various authors [50,51].

In the elderly population of the present study, LOHS did not change significantly between 2016 and 2022, although it was longer in elderly patients with RSV infection. Ackerson et al. found that patients with RSV infection were more likely to require a hospital stay ≥7 days and ICU admissions than those with influenza [52]. Along the same lines, we showed that invasive and non-invasive mechanical ventilation were more frequently coded in individuals with RSV infection than in those without RSV infection. However, no differences were found in the proportion requiring ICU admission, IHM, or costs. In this regard, previous studies reported the severity of clinical outcomes of RSV infection among older adults in different settings [47].

The mortality of RSV infection is an important component of disease burden [53]. Epidemiological data indicate that RSV infection is a significant contributor to morbidity and mortality in adults, with a disease burden at least comparable to that of seasonal influenza [6].

We found an increase in the number of deaths from 2019 to 2022, although this increase was not statistically significant, likely owing to the small number of cases. In fact, RSV itself was not significantly associated with IHM when the entire population of elderly patients with asthma was analyzed. Factors associated with IHM in these patients were advanced age, congestive heart failure, COVID-19, and pneumonia. Lee et al. also examined the role of pneumonia in adult RSV infections and found that bacterial superinfection was a significant contributor to mortality [15].

The main strength of our study lies in its very large sample size, as hospitalizations with RSV infection were identified and analyzed in a population-wide dataset of all Spanish hospitals, both public and private. Moreover, we included different age groups, while other studies mainly focused on children. Notwithstanding, our study has several limitations that are inherent to its retrospective design, which was based on discharge codes. First, because RSV diagnosis is based on an administrative database, there is potential for underdiagnosis/underdetection of RSV. For example, Prasad et al. [54] carried out prospective surveillance in hospitalized adults with underlying conditions (including asthma) and detected the presence of RSV infection in 173/2238 (7.7%) of patients with asthma, compared with 0.17–1.01% of RSV in adults in the present study. Falsey et al. [14] also reported a higher prevalence of RSV (7.2%) among patients with asthma admitted to the hospital. High prevalences of viruses (including RSV) have also been reported in children with asthma exacerbations [55]. Many children are discharged before laboratory test results for RSV infection are available, or they are simply coded as having “acute bronchiolitis” even though they are RSV-positive [6]. Therefore, the use of more restrictive codes to collect only confirmed RSV-positive cases could have resulted in missing a significant proportion of RSV-related infections [56]. Furthermore, we do not have confirmation that the group of patients defined as “without RSV” were tested negative, which also represents a limitation and may affect the observed results. Second, the use of this administrative database to assess patients with asthma diagnosis could also potentially lead to misdiagnoses, since the diagnosis of asthma in young children, particularly before one year of age, is difficult to establish, and because bronchiolitis presents similar symptoms. However, other studies that have been conducted in patients with asthma and that have used administrative databases have included all patients, without excluding children less than one year old [57,58,59]. Third, another limitation of this study is that we did not have laboratory or drug administration data. Fourth, all patients coded with RSV infection, and community-acquired and nosocomial infections, were included in the study. We decided to analyze all patients together, even if the infection was acquired in the hospital, because only 12 children (0.7%) and 36 elderly patients (1.5%) had a code for nosocomial infection. It is well known that hospital-acquired infections tend to be more severe than community-acquired infections, since they occur in patients with underlying conditions, patients may already be admitted to the ICU, and length of stay is usually more prolonged. Future studies, with a larger number of nosocomial infections, should compare these two types of RSV infections. Finally, since we used hospitalization data, we were only able to quantify the burden of RSV infection in hospitalized patients. To obtain a complete picture of the burden of medically managed RSV infections in Spain, outpatient treatment data are also relevant.

## 5. Conclusions

Our results highlight the impact of RSV infection in children and elderly patients hospitalized with asthma. It is necessary to explore and evaluate strategies for improved surveillance, prophylaxis, and management of RSV infection in these populations with the aim of reducing disease burden.

## Figures and Tables

**Table 1:** Characteristics of children (0–15 years) and elderly patients (65 year or over) hospitalized with a diagnosis of asthma and respiratory syncytial virus infection (RSVI) in Spain (2016–2022).

Children (0–15 Years)		2016	2017	2018	2019	2020	2021	2022	*p* for Time Trend
Children hospitalized with asthma, n		8419	8043	8136	7194	5045	5286	6963	NA
Children hospitalized with asthma and RSVI, n (%)		121 (1.44)	172 (2.14)	198 (2.43)	308 (4.28)	119 (2.36)	295 (5.58)	515 (7.4)	<0.001
Sex, n (%)	Boys	65 (53.72)	102 (59.3)	127 (64.14)	182 (59.09)	58 (48.74)	170 (57.63)	297 (57.67)	0.206
	Girls	56 (46.28)	70 (40.7)	71 (35.86)	126 (40.91)	61 (51.26)	125 (42.37)	218 (42.33)	
Age, mean (SD)		2.09 (2.41)	2.22 (2.54)	1.91 (2.09)	2.04 (2.15)	3.18 (3.61)	2.65 (2.38)	3.43 (3.16)	<0.001
Age groups, n (%)	0–1 years	72 (59.5)	81 (47.09)	110 (55.56)	159 (51.62)	50 (42.02)	92 (31.19)	146 (28.35)	<0.001
	2–5 years	36 (29.75)	77 (44.77)	76 (38.38)	128 (41.56)	48 (40.34)	178 (60.34)	278 (53.98)	
	6–15 years	13 (10.74)	14 (8.14)	12 (6.06)	21 (6.82)	21 (17.65)	25 (8.47)	91 (17.67)	
Invasive mechanical ventilation, n (%)	Yes	2 (1.65)	0 (0)	1 (0.51)	2 (0.65)	1 (0.84)	2 (0.68)	1 (0.19)	0.490
Non-invasive mechanical ventilation, n (%)	Yes	8 (6.61)	4 (2.33)	5 (2.53)	19 (6.17)	4 (3.36)	16 (5.42)	22 (4.27)	0.244
Admission to ICU, n (%)	Yes	11 (9.09)	8 (4.65)	14 (7.07)	26 (8.44)	7 (5.88)	27 (9.15)	34 (6.6)	0.525
Severe cases *, n (%)	Yes	15 (12.4)	11 (6.4)	16 (8.08)	34 (11.04)	10 (8.4)	34 (11.53)	45 (8.74)	0.389
LOHS, median (IQR)		5 (4)	4 (4)	4.5 (3)	4 (3)	5 (3)	4 (3)	4 (3)	0.001
Costs in euros, mean (SD)		3181 (2923)	2754 (1195)	2735 (1541)	2811 (2779)	3159 (2277)	3048 (1431)	2909 (1081)	0.118
**Elderly (65 Years and over)**									
Elderly hospitalized with asthma, n		55,272	64,090	69,941	71,946	64,229	68,379	78,090	NA
Elderly hospitalized with asthma and RSVI, n (%)		93 (0.17)	204 (0.32)	291 (0.42)	494 (0.69)	405 (0.63)	123 (0.18)	779 (1.01)	<0.001
Sex, n (%)	Men	20 (21.51)	30 (14.71)	44 (15.12)	74 (14.98)	64 (15.8)	20 (16.26)	134 (17.2)	0.719
	Women	73 (78.49)	174 (85.29)	247 (84.88)	420 (85.02)	341 (84.2)	103 (83.74)	645 (82.8)	
Age, mean (SD)		78.68 (6.83)	79.88 (7.91)	79.98 (7.55)	80.35 (7.87)	80.78 (7.42)	81.6 (8.04)	80.79 (8.11)	0.006
Age groups, n (%)	65–74 years	25 (26.88)	62 (30.39)	77 (26.46)	135 (27.33)	88 (21.73)	29 (23.58)	201 (25.8)	0.003
	75–84 years	49 (52.69)	80 (39.22)	127 (43.64)	179 (36.23)	172 (42.47)	40 (32.52)	283 (36.33)	
	85+ years	19 (20.43)	62 (30.39)	87 (29.9)	180 (36.44)	145 (35.8)	54 (43.9)	295 (37.87)	
Invasive mechanical ventilation, n (%)	Yes	4 (4.3)	6 (2.94)	7 (2.41)	9 (1.82)	4 (0.99)	2 (1.63)	9 (1.16)	0.169
Non-invasive mechanical ventilation, n (%)	Yes	1 (1.08)	9 (4.41)	16 (5.5)	20 (4.05)	23 (5.68)	10 (8.13)	33 (4.24)	0.228
Admission to ICU, n (%)	Yes	11 (11.83)	16 (7.84)	12 (4.12)	26 (5.26)	14 (3.46)	7 (5.69)	21 (2.7)	<0.001
IHM, n (%)	Yes	2 (2.15)	12 (5.88)	19 (6.53)	25 (5.06)	23 (5.68)	12 (9.76)	39 (5.01)	0.278
LOHS, median (IQR)		7 (7)	7 (6)	7(6)	7 (6)	8 (7)	8 (7)	7 (6)	0.097
Costs in euros, mean (SD)		4453 (6741)	3808 (3622)	3700 (5311)	3715(4222)	4286 (4932)	4449 (7393)	3856 (4596)	0.367

* Severe cases included those who were admitted to ICU and/or required mechanical ventilation. SD: Standard deviation. ICU: Intensive care unit. IHM: In-hospital mortality. IQR: Interquartile range. LOHS: Length of hospital stay. NA: Not available.

**Table 2:** Characteristics and hospital outcomes of children (0–15 years) and elderly patients (65 year or over) hospitalized with a diagnosis of asthma and respiratory syncytial virus infection (RSVI) and age–sex-matched controls without RSVI in Spain (2016–2022).

Children (0–15 Years)		RSVI	No RSVI	*p*
Number of children		1727	1727	NA
Sex, n (%)	Boys	1000 (57.9)	1000 (57.9)	NA
	Girls	727 (42.1)	727 (42.1)	
Age, mean (SD)		2.65 (2.75)	2.65 (2.75)	NA
Age groups, n (%)	0–1 years	709 (41.05)	709 (41.05)	NA
	2–5 years	821 (47.54)	821 (47.54)	
	6–15 years	197 (11.41)	197 (11.41)	
Invasive mechanical ventilation, n (%)	Yes	9 (0.52)	5 (0.29)	0.284
Non-invasive mechanical ventilation, n (%)	Yes	78 (4.52)	42 (2.43)	0.001
Admission to ICU, n (%)	Yes	127 (7.35)	99 (5.73)	0.054
Severe cases *, n (%)	Yes	165 (9.55)	115 (6.66)	0.002
LOHS, median (IQR)		4 (3)	3 (2)	<0.001
Costs in euros, mean (SD)		2916 (1858)	2618 (1079)	<0.001
**Elderly (65 Years and over)**				
Number of elderly hospitalized		2389	2389	NA
Sex, n (%)	Men	386 (16.16)	386 (16.16)	NA
	Women	2003 (83.84)	2003 (83.84)	
Age, mean (SD)		80.48 (7.82)	80.48 (7.82)	NA
Age groups, n (%)	65–74 years	617 (25.83)	617 (25.83)	NA
	75–84 years	930 (38.93)	930 (38.93)	
	85+ years	842 (35.24)	842 (35.24)	
Invasive mechanical ventilation, n (%)	Yes	41 (1.72)	22 (0.92)	0.016
Non-invasive mechanical ventilation, n (%)	Yes	112 (4.69)	50 (2.09)	<0.001
Admission to ICU, n (%)	Yes	107 (4.48)	94 (3.93)	0.349
IHM, n (%)	Yes	132 (5.53)	161 (6.74)	0.080
LOHS, median (IQR)		7 (7)	6 (7)	<0.001
Costs in euros, mean (SD)		3930 (4881)	4049 (3121)	0.107

* Severe cases included those who were admitted to ICU and/or required mechanical ventilation. SD: Standard deviation. ICU: Intensive care unit. IHM: In-hospital mortality. IQR: Interquartile range. LOHS: Length of hospital stay. NA: Not available.

**Table 3:** Underlying conditions and related diagnoses among children hospitalized with a diagnosis of asthma and respiratory syncytial virus infection (RSVI) and age–sex-matched controls without RSVI in Spain (2016–2022).

	RSVI	No RSVI	*p*
Congestive heart failure, n (%)	2 (0.12)	0 (0)	0.157
Chronic renal disease, n (%)	1 (0.06)	2 (0.12)	0.564
Diabetes, n (%)	2 (0.12)	2 (0.12)	1.000
Liver disease, n (%)	0 (0)	1 (0.06)	0.317
Cancer, n (%)	4 (0.23)	5 (0.29)	0.739
Acute bronchitis, n (%)	194 (11.23)	93 (5.39)	<0.001
Bronchiolitis, n (%)	194 (11.23)	18 (1.04)	<0.001
Influenza, n (%)	24 (1.39)	46 (2.66)	0.008
COVID-19, n (%)	14 (0.81)	29 (1.68)	0.021
Pneumonia, n (%)	77 (4.46)	143 (8.28)	<0.001
Obesity, n (%)	9 (0.52)	16 (0.93)	0.160
Dyspnea, n (%)	27 (1.56)	17 (0.98)	0.129
Otitis, n (%)	61 (3.53)	34 (1.97)	0.005

**Table 4:** Underlying conditions and related diagnoses among elderly subjects hospitalized with a diagnosis of asthma and respiratory syncytial virus infection (RSVI) and age–sex-matched controls without RSV infection in Spain (2016–2022).

		RSVI	No RSVI	*p*
CCI, n (%)	0	876 (36.67)	791 (33.11)	0.003
	1–2	1059 (44.33)	1057 (44.24)	
	3+	454 (19)	541 (22.65)	
CCI, mean (SD)		1.35 (1,53)	1.56 (1.72)	<0.001
Congestive heart failure, n (%)	Yes	630 (26.37)	580 (24.28)	0.096
Myocardial infarction, n (%)	Yes	16 (0.67)	33 (1.38)	0.015
Chronic renal disease, n (%)	Yes	404 (16.91)	411 (17.2)	0.788
Depression, n (%)	Yes	148 (6.2)	182 (7.62)	0.052
Diabetes, n (%)	Yes	677 (28.34)	656 (27.46)	0.498
Liver disease, n (%)	Yes	82 (3.43)	107 (4.48)	0.064
Peripheral vascular disease, n (%)	Yes	59 (2.47)	89 (3.73)	0.012
Cerebrovascular disease, n (%)	Yes	97 (4.06)	156 (6.53)	<0.001
Cancer, n (%)	Yes	121 (5.06)	215 (9)	<0.001
COPD, n (%)	Yes	277 (11.59)	208 (8.71)	0.001
Emphysema, n (%)	Yes	24 (1)	21 (0.88)	0.653
Bronchiectasis, n (%)	Yes	133 (5.57)	117 (4.9)	0.299
Acute bronchitis, n (%)	Yes	406 (16.99)	97 (4.06)	<0.001
Bronchiolitis, n (%)	Yes	72 (3.01)	1 (0.04)	<0.001
Influenza, n (%)	Yes	76 (3.18)	52 (2.18)	0.032
COVID-19, n (%)	Yes	32 (1.34)	101 (4.23)	<0.001
Pneumonia, n (%)	Yes	178 (7.45)	199 (8.33)	0.260
Obesity, n (%)	Yes	452 (18.92)	427 (17.87)	0.351
OSA, n (%)	Yes	248 (10.38)	257 (10.76)	0.672

CCI: Charlson Comorbidity Index. COPD: Chronic obstructive pulmonary disease. OSA: Obstructive sleep apnea.

**Table 5:** Factors associated with severity among children with a diagnosis of asthma according to the presence of respiratory syncytial virus infection (RSVI) in Spain (2016–2022).

		RSVI	No RSVI	All Children
		OR (95% CI)	OR (95% CI)	OR (95% CI)
Age groups, n (%)	0–1 years	NS	NS	1
	2–5 years	NS	NS	NS
	6–15 years	NS	NS	1.48 (1.04–2.16)
Acute bronchitis	Yes	NS	2 (1.02–3.92)	1.49 (1.01–2.21)
Bronchiolitis	Yes	NS	7.4 (2.44–22.5)	NS)
Influenza	Yes	3.65 (1.46–9.1)	NS	NS
COVID-19	Yes	NS	3.3 (1.29–8.49)	NS
Pneumonia	Yes	1.85 (1.02–3.55)	NS	NS
RSVI	Yes	NA	NA	1.44 (1.11–1.86)

OR: Odds ratio. CI: Confidence interval. NS: Not significant. NA: Not applicable.

**Table 6:** Factors associated with in-hospital mortality among elderly patients with a diagnosis of asthma according to the presence of respiratory syncytial virus infection (RSVI) in Spain (2016–2022).

		RSVI	No RSVI	All Elderly
		OR (95% CI)	OR (95% CI)	OR (95% CI)
Age groups	65–74 years	1	1	1
	75–84 years	4.09 (2.04–8.2)	2.35 (1.22–4.52)	3.17 (1.98–5.1)
	85+ years	7.39 (3.57–15.29)	6.2 (3.27–11.77)	6.75 (4.18–10.9)
Congestive heart failure	Yes	2.2 (1.51–3.22)	2.21 (1.53–3.19)	2.19 (1.69–2.84)
Myocardial infarction	Yes	NS	2.89 (1.17–7.14)	1.73 (0.78–3.86)
Chronic renal disease	Yes	NS	1.53 (1.03–2.26)	1.15 (0.85–1.54)
Cerebrovascular disease	Yes	NS	NS	1.68 (1.07–2.63)
Cancer	Yes	NS	NS	2.62 (1.75–3.92)
COVID-19	Yes	2.82 (1.18–8.53)	3.31 (1.8–6.11)	3.06 (1.81–5.18)
Pneumonia	Yes	1.89 (1.11–2.99)	1.76 (1.13–2.95)	1.80 (1.18–2.49)
RSVI	Yes	NA	NA	0.88 (0.68–1.15)

OR: Odds ratio. CI: Confidence interval. NS: Not significant. NA: Not applicable.

## Data Availability

According to the contract signed with the Spanish Ministry of Health and Social Services, which provided access to the databases from the Spanish National Hospital Database, we cannot share the databases with any other investigator, and we have to destroy the databases once the investigation has concluded. Consequently, we cannot upload the databases to any public repository. However, any investigator can apply for access to the databases by filling out the questionnaire available at https://www.sanidad.gob.es/estadEstudios/estadisticas/estadisticas/estMinisterio/SolicitudCMBD.htm (accessed on 20 June 2024). All other relevant data are included in the paper.

## Supplementary Materials

The following supporting information can be downloaded at https://www.mdpi.com/article/10.3390/v16111749/s1: Table S1: International Classification of Diseases, 10th Revision (ICD-10) codes used in this investigation.
