# Supplementary material for: Burden of Respiratory Syncytial Virus Infection in Children and Older Patients Hospitalized with Asthma: A Seven-Year Longitudinal Population-Based Study in Spain
Source: Viruses. 2024 Nov 7;16(11):1749. doi: 10.3390/v16111749 (PMC11599133; doi:10.3390/v16111749)
Supplement: Supplementary file 1 [file viruses-16-01749-s001.zip › viruses-3240177-supplementary.pdf]

Table S1. International Classification of Diseases 10th Revision (ICD10) codes used in this investigation.

**Diagnosis or procedure**	**IC10 codes**
Syncytial respiratory virus infection	J12.1, J20.5, J21.0, B97.4
Asthma	J45
Congestive Heart Failure	I50
Myocardial infarction	I21, I22
Chronic Renal disease	N18
Depression	F32
Diabetes	E10, E11
Liver disease	K72 to K77
Peripheral Vascular Disease	I71, I790, I739, R02, Z958, Z959
Cerebrovascular Disease	I60, I61, I62, I63, I65, I66,G450, G451, G452, G458, G459, G46, I64, G454, I670, I671, I672, I674, I675, I676, I677 I678, I679, I681, I682, I688, I69
Cancer	C00, C26, C30, C34, C37, C41, C43, C45., C58, C60, C76, C81, C85, C88, C90, C97, C77, C80
COPD	J41, J42, J43, J44
Emphysema	J43
Bronchiectasis	J47
Acute bronchitis	J20
Bronchiolitis	J21
Influenza	J09-J11
COVID 19	B97.29 U07.1
Pneumonia	J13 to J18 and J95.851
Obesity	E66.09, E66.1 E66.3, E66.8 E66.9 E66.2, E66.01
Dyspnea:	R06.0
Otitis:	H66.9
OSA	G47.3-G473.9
Invasive Mechanical ventilation	5A1935Z 5A1945Z, 5A1955Z,
Non-Invasive Mechanical ventilation	5A09357, 5A09457, 5A09557

COPD Chronic Obstructive Pulmonary Disease. OSA Obstructive Sleep Apnea
